# Preparation of PLGA/MWCNT Composite Nanofibers by Airflow Bubble-Spinning and Their Characterization

**DOI:** 10.3390/polym10050481

**Published:** 2018-04-28

**Authors:** Yue Fang, Fujuan Liu, Lan Xu, Ping Wang, Jihuan He

**Affiliations:** National Engineering Laboratory for Modern Silk, College of Textile and Engineering, Soochow University, 199 Ren-ai Road, Suzhou 215123, China; yfang5279@stu.suda.edu.cn (Y.F.); lanxu@suda.edu.cn (L.X.); pingwang@suda.edu.cn (P.W.); hejihuan@suda.edu.cn (J.H.)

**Keywords:** airflow bubble-spinning, composite nanofibers, multi-walled carbon nanotubes, PLGA

## Abstract

Poly(lactic-*co*-glycolic acid) (PLGA)/multi-walled carbon nanotube (MWCNT) composite nanofibers have been successfully fabricated via airflow bubble-spinning. In this work, a systematic study of the effects of solution concentration, relative humidity (RH), and composition on the morphology of PLGA nanofibers is reported. By comparing the distribution of fiber diameter, we found that the spinning effect was the best when the temperature was kept at 25 °C, the collecting distance 18 cm, the concentration 8 wt %, and the relative humidity 65%. MWCNTs used as added nanoparticles were incorporated into the PLGA nanofibers. The volatile solvents were used to achieve the purpose of producing nanoporous fibers. Besides, the rheological properties of solutions were studied and the PLGA or PLGA/MWCNT composite nanofibers with a nanoporous structure were also completely characterized using scanning electron microscope (SEM), a thermogravimetric analyzer(TGA), X-ray diffraction(XRD) and Fourier-transform infrared (FTIR) spectroscopy. In addition, we compared the mechanical properties of the fibers. It was found that the addition of MWCNTs significantly enhanced the tensile strength and elasticity of composite nanofibers without compromising the nanoporous morphology. The results showed that the breaking strength of the composite fiber bundle was three times as strong as the pure one, and the elongation at the break was twice as great. This work provided a novel technique successfully not only to get rid of the potential safety hazards caused by unexpected static but also prepare oriented nanoporous fibers, which would demonstrate an impressive prospect for the fields of adsorption and filtration.

## 1. Introduction

Electrospinning has been recognized as the best simple and efficient technique for the production of polymer nanofibers. Its main working principle is to use electric field force to overcome the surface tension of the Taylor cone of solution or melt in the high voltage electric field environment, and the spinning liquid that undergoes charge transfer is stretched and refined to form nanofibers. Due to the high surface area, high surface activity and high surface energy, electrospun nanofibers can be used in a wide variety of applications such as nonwoven fabrics [1], sensorics [2], photonics [3], filtration [4], composites [5], wound dressing [6], tissue engineering [7], fuel cells [8] and so on. However, due to the low production volume of conventional single-needle electrostatic spinning, the application of nanofibers in commercial production is inhibited, and its yield is usually 0.01–0.1 g/h [9]. Therefore, a new spinning process, bubble electrospinning, came into being.

Bubble electrospinning [10,11,12,13,14,15,16], as the initial stage of the bubble spinning process, was first invented in 2007 by Professor Jihuan He and his team (CHN Patent No. ZLGL2007030001). Similar to the electrospinning process, bubble electrospinning is also performed under a high-voltage electric field environment. It utilizes electric field force to overcome the surface tension of the polymer or melt gas bubbles and the film, instead of the traditional surface tension of electrospinning Taylor cones. Then the bubbles and the film are ruptured, eventually becoming nanofibers due to the electric field force. Compared with traditional single-needle spinning, the bubble electrospinning process is not only simpler and more effective, but also can significantly increase the production of nanofibers.

However, whether it is bubble electrospinning or traditional electrostatic spinning, thousands or even tens of thousands of volts are needed to provide the spinning power. Such a high voltage also brings an inevitable huge safety hazard, which challenges both laboratory and industrial production. The airflow bubble-spinning method [17,18] is used in this paper, which utilizes an external airflow with a certain temperature and speed for the surface tension of the film, polymer, and melt bubbles. This is different from the electric field force in bubble electrospinning. The airflow promotes the bursting of the bubble, and due to the bubble bursting, jets will be produced, stretching with the solvent evaporation and jet curing, and nanofibers will finally be formed on the collecting plate. This not only has the advantage of a high yield from the bubble spinning method, but also avoids the threat of high voltage static electricity.

The spinning material PLGA is a widely used biodegradable and biocompatible polymer. In recent years, with the development of electrospinning technology, PLGA nanofiber mats prepared by electrospinning have been widely utilized in the medical field, such as surgery, drug release devices and tissue engineering scaffolds [19,20,21,22]. In recent years, people’s attention has shifted from pure PLGA to nanocomposite materials, as for PLGA/MWCNT composite nanofibers. Qi and Rui-ling et al. have studied the release of doxorubicin from electrospun MWCNT/PLGA hybrid nanofibers [23]. Wang and Jing et al. have studied the effect of plasma treated PLGA/MWCNT-COOH composite nanofibers on nerve cell behavior [24]; and the effect of the porous microstructures of poly(lactic acid-*co*-glycolic acid)/carbon nanotube composites on the growth of fibroblast cells has been studied by Fujuan Liu et al. [25].

Carbon nanotubes (CNTs) is one of the most lightweight materials to date, and it has excellent electrical conductivity and thermal conductivity. As a new generation of high performance reinforcements, it has unpredictable potential for development. In particular, the continuous carbon nanotube composites that are made of continuous carbon nanotubes fibers and carbon nanotubes (CNTs) have been recognized as epoch-making third-generation advanced composites [26,27,28]. Moreover, there has been much research focused on the biological applications of CNTs at the cellular level recently. These studies clearly demonstrate that CNTs can be used as one of the most promising nanofibers to mimic the extracellular matrix. Early in this development process, Liu and her team successfully incorporated multi-walled carbon nanoparticles into polymers for electrospinning. The composite nanofibers are uniform and have excellent mechanical properties [29].

CNTs have good heat transfer performance and a large aspect ratio, thus the heat exchange performance in the length direction is high and the heat exchange performance in the vertical direction is relatively low. With proper orientation, the carbon nanotubes can synthesize highly anisotropic heat conduction materials. In addition, carbon nanotubes have high thermal conductivity. As long as the carbon nanotubes are doped in the composite material, the thermal conductivity of the composite material may be greatly improved [30].

This article utilized airflow bubble-spinning without high-voltage electrostatic hazards to fabricate PLGA/MWCNT composite nanofibers. Nanofibers with porous structure were also obtained. Additionally, we show that multi-walled carbon nanotubes (MWCNTs) can be incorporated into PLGA nanofibers under optimal spinning conditions. The composite nanofibers were expected to have superior mechanical properties when compared to the pure PLGA nanofibers. These PLGA or PLGA/MWCNT composite nanofibers were also characterized by using scanning electron microscopy (SEM), thermogravimetric analyzer (TGA), X-ray diffraction (XRD) and Fourier-transform infrared (FTIR) spectroscopy. The PLGA or PLGA/MWCNTs composite nanofibers should be especially useful for the development of adsorption and filtration materials in the future.

## 2. Experimental

### 2.1. Materials

The poly(lactide-*co*-glycolide) acid (PLGA, *M*_w_ =113,000 g/mol) used in this experiment was purchased from Jinan Daigang Biological Engineering Co. Ltd. (Jinan, China). Chloroform (CF) (analytical reagent) was purchased from Shanghai Lingfeng Chemical Reagent Co. Ltd. (Shanghai, China). All chemicals were used as received without further purification.

Multi-walled carbon nanotubes (MWCNTs) (10–20nm in diameter; 10–30μm in length) were purchased from Beijing Deke Island Gold Co. Ltd. (Beijing, China).

PLGA was dissolved in chloroform and then a magnetic stirrer (HJ-6A Gongyi to China Instrument Co. Ltd., Gongyi, China) was used to stir the obtained mixture for about 1.5 h at room ambient temperature, to prepare transparent solutions with concentrations of 5, 6, 7, 8 and 10 wt %, respectively. A total of 0.1 wt % MWCNTs was added into the 8 wt % solution, and then the mixed solution was subjected to ultrasonic oscillations of not less than thirty minutes using an ultrasonic cleaner (SL-5200DT, Nanjing Shunliu Instrument Co. Ltd., Nanjing, China).

### 2.2. The Apparatus of Airflow Bubble-Spinning

**Figure 1:**
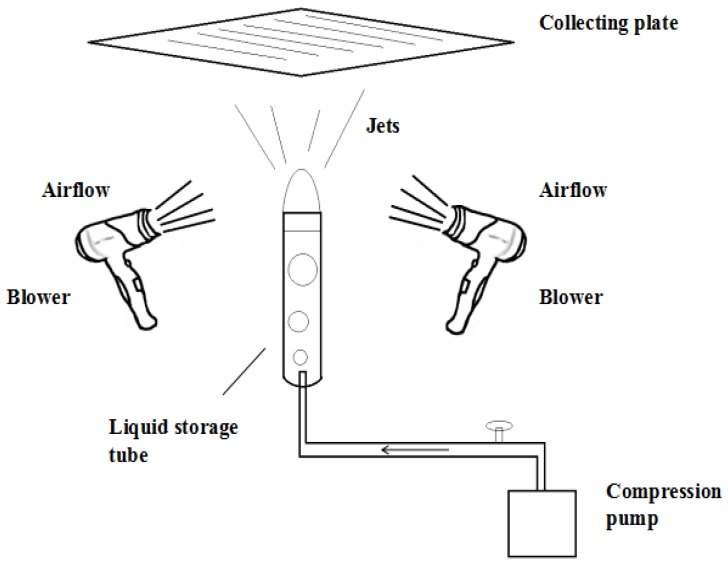
The schematic of the airflow bubble-spinning.

According to the principle of the airflow bubble-spinning method described above, the experimental set-up of the airflow bubble-spinning is indicated in Figure 1. As shown, the device is mainly composed of a foaming device, collecting device, and a blowing device. The foaming device consists of a liquid storage tube and a small air pump that can provide airflow for the generation of air bubbles. The blowing device consists of two blowers. The diameter of the liquid storage tube used in this experiment was 10 mm and the airflow rate used in this test was 500 L/min. The collecting distance *D* was 18 cm.

**Figure 2:**
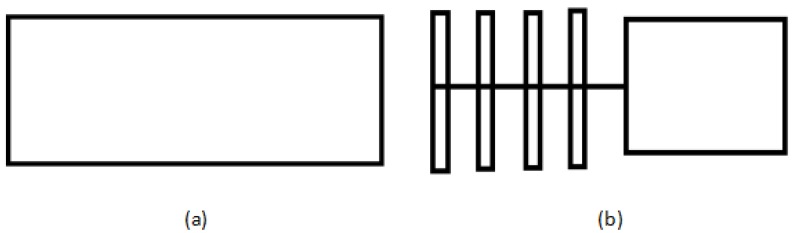
Collecting device schematic: (**a**)the collecting board for a full area, and (**b**) row column collecting device.

Figure 2 shows the collecting device schematic: (a) as the full collecting board is to receive nanofibers; and (b) as the row column collecting device is to receive nanofibers for the mechanical behavior test.

When we used the new airflow bubble-spinning method to spin, it was always easy to form large droplets on the collecting device. The droplets would fix the fibers and fiber bundles on the tin foil so that the spinning samples ware difficult to peel off from the foil. This is a big problem for sampling to test the mechanical properties. The full collecting board was used to collect the nanofibers for SEM characterization and the row column collecting device was used to intercept the fiber stretched in the air by the column net on the collecting device for the tensile performance test.

### 2.3. The Process of Airflow Bubble-Spinning

When the airflow bubble-spinning experiments were carried out, the prepared spinning solution was loaded into the liquid storage tube. The compressed air produced by the air pump causes the spinning solution to form continuous single bubble, and the blower blows out the airflow, causing the bubbles become large and eventually rupture. At the same time, the jets generated by the bubble are stretched. Accompanied by solvent evaporation, the jets will solidify and the PLGA nanoporous fibers will be deposited on the collecting plate. This process was repeated until the polymer bubble in the tube could not be generated. The relative humidity used in the experimental process was 65%.

### 2.4. Measurement and Characterization

The morphology of the PLGA or PLGA/MWCNT composite nanofibers was characterized using a scanning electron microscope (Hitachi S-4800, Tokyo, Japan). The samples were sputter-coated with a gold film before measurements. The fiber diameters were determined using Image J software (developed by the U.S. National Institutes of Health, Bethesda, MD, USA). At least 20 nanofibers in different SEM images were analyzed for each sample. 

Rheological studies were run on a rheometer (TA Instruments, AR2000, New Castle, DE, USA) with a 40 mm cone plate (Ti, 40/1°). The normal force applied to the sample during the lowering of the top plate was limited to 0.1 N. The shear rate was linearly increased from 0.1 to 1000 1/s at 25 °C. All rheological measurements were repeated on at least two different samples.

The thermogravimetric analysis was carried out using the thermo-differential thermal analyzer (TG/DTA, PerkinElmer, Waltham, MA, USA). The samples were heated at 20 °C/min in a nitrogen atmosphere with a temperature control range from room temperature to 600 °C.

X-ray diffraction was performed by a fully automatic X-ray diffractometer (X’Pert-Pro MRD, Philips, Amsterdam, The Netherlands). The test conditions were CuKα rays, X-ray wavelength λ = 1.54076 Å, and a super-detection counter recording the X-diffraction intensity curve where 2θ is between 5° and 60°.

FTIR was measured using the infrared spectrometer (Nicolet 5700, Thermo Nicolet Company, Waltham, MA, USA). In this test, we needed to collect 1–2 mg fiber samples and cut them into powder. In order to crush the samples to the micron level, about 200 mg KBr powder were added to the samples. The mixture was fully ground and then became thin slices through the compression method. After the success of the tablet presses, it was again necessary to dry to reduce the interference of the water vapor absorption peak. Finally, the drying tablets were put into the infrared spectrometer for testing.

The mechanical properties were determined by equilibrating the fiber bundle for 24 h at a constant temperature and humidity (temperature 20 ± 2 °C, relative humidity 65 ± 2%) and then performing the test on an Instron 3365 Universal Power Meter (Instron, Norwood, MA, USA). The test conditions were a clamping length of 20 mm, with a pre-tension of 0.2 cN and a tensile rate of 10 mm/min, respectively. 

## 3. Results and Discussion

### 3.1. Effect of the Spinning Concentration on the Morphology of PLGA Fibers

**Figure 3:**
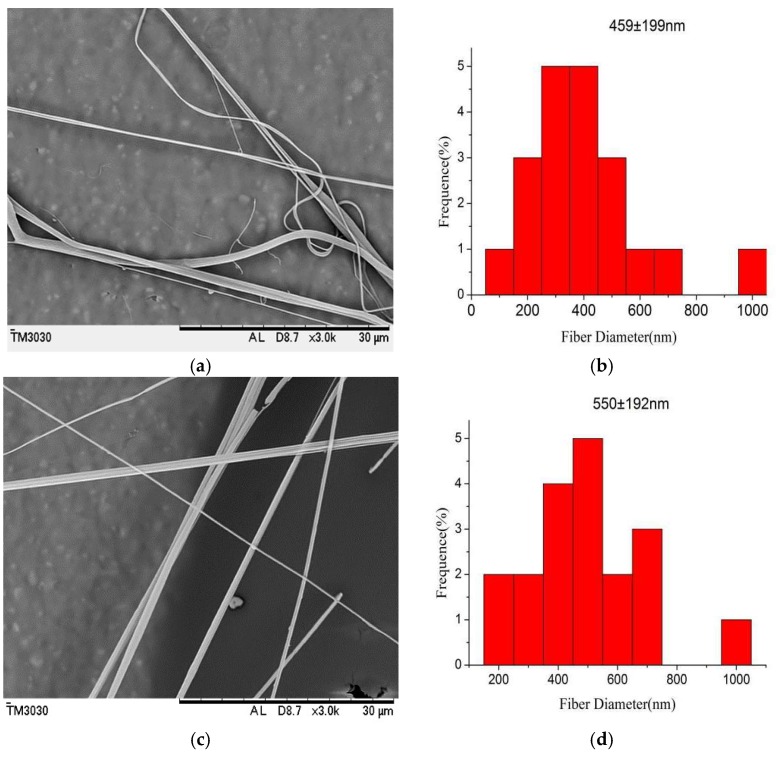

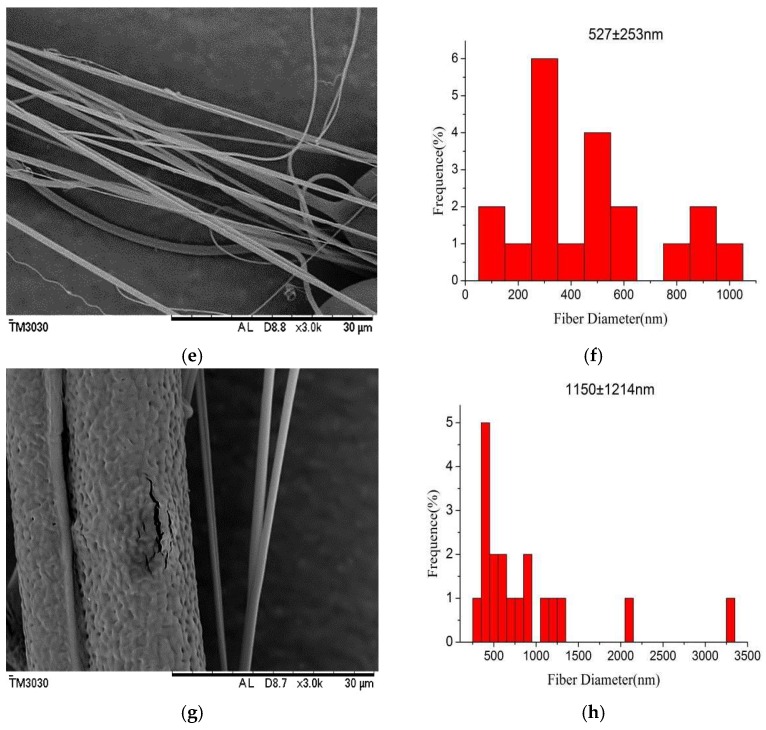
SEM micrographs and the diameter distribution histograms of nanoporous fibers via the airflow bubble-spun PLGA solutions with concentrations of (**a**) 6 wt %; (**c**) 7 wt %; (**e**) 8 wt %; (**g**) 10 wt % (temperature: 25 °C, collecting distance: 18 cm, relative humidity: 65%); (**b**,**d**,**f**,**h**) show the diameter distribution histograms of PLGA nanoporous fibers corresponding to (**a**,**c**,**e**,**g**), respectively. The diameter distribution histogram data are representative of independent experiments and all data are given as means ± SD (*n* = 20).

Fiber morphology is closely associated with solution concentrations. In the ideal state, the chloroform solvent used in this experiment is highly volatile. When the solvent evaporation rate is greater than the jetting, holes will form on the fibers, which are nanoporous. Figure 3 shows the SEM micrographs and the diameter distribution histograms of the nanoporous fibers obtained with airflow bubble-spun PLGA solutions with different concentrations (from 6 to 10 wt %). When the solution concentration was lower than 5 wt %, there were no effective nanofibers obtainedon the tin foil paper (not shown in Figure 3). When the concentrations were kept at 6 and 7 wt %, the fibers were obtained on the collecting plate, but the porous effect was not obvious (Figure 3a,c). However, if the concentrations were increased to 8 and 10 wt %, the fiber surface showed clear porosity (Figure 3e,g).

**Figure 4:**
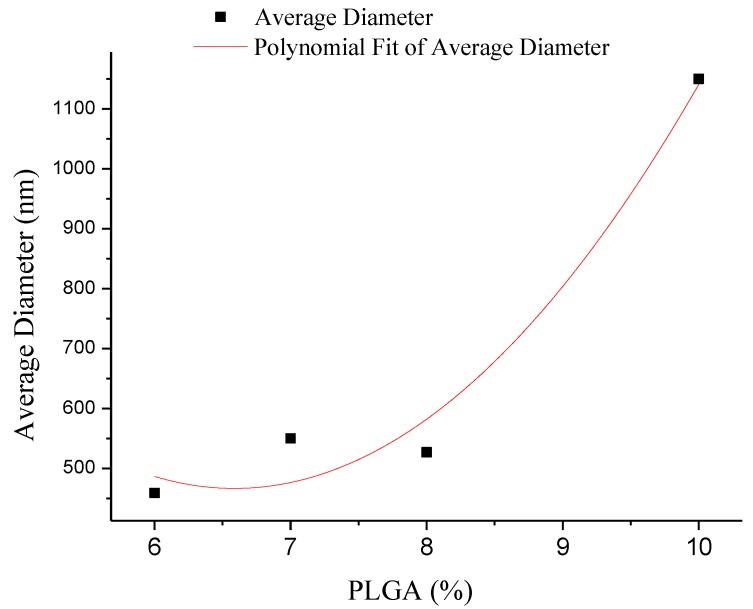
Average diameterof composite nanofibers with the different concentrations.

The fiber diameters were also measured to be 459, 550, 527 and 1150 nm at concentrations of 6, 7, 8 and 10 wt %, respectively. The diameter of the fibers increased non-linearly with the concentration (Figure 4), which is consistent with results reported in the literature [29]. The diameter distribution histograms of the nanofibers formed under various concentrations show that the formed PLGA fibers are relatively uniform (Figure 3b,d,f,h). 

### 3.2. Effect of Relative Humidity on the Morphology of PLGA Fibers

**Figure 5:**
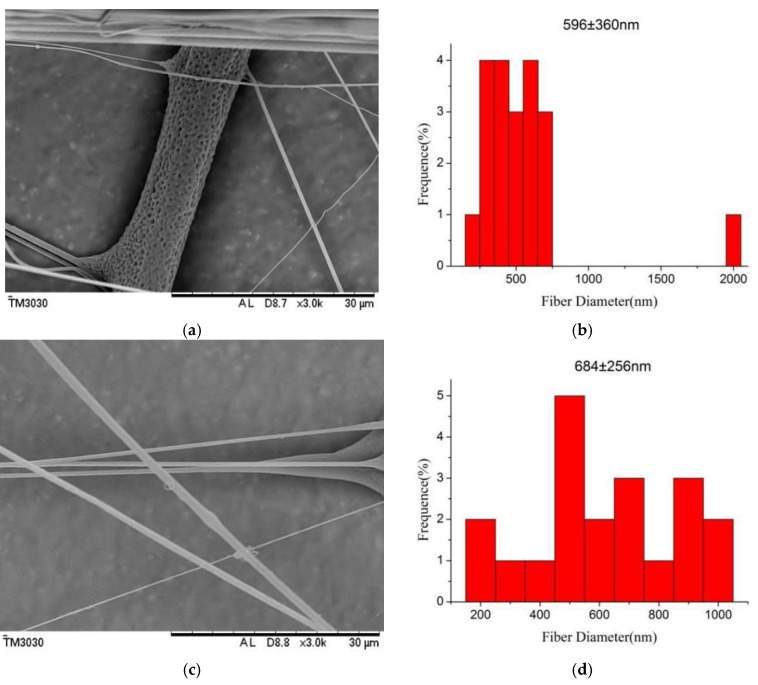

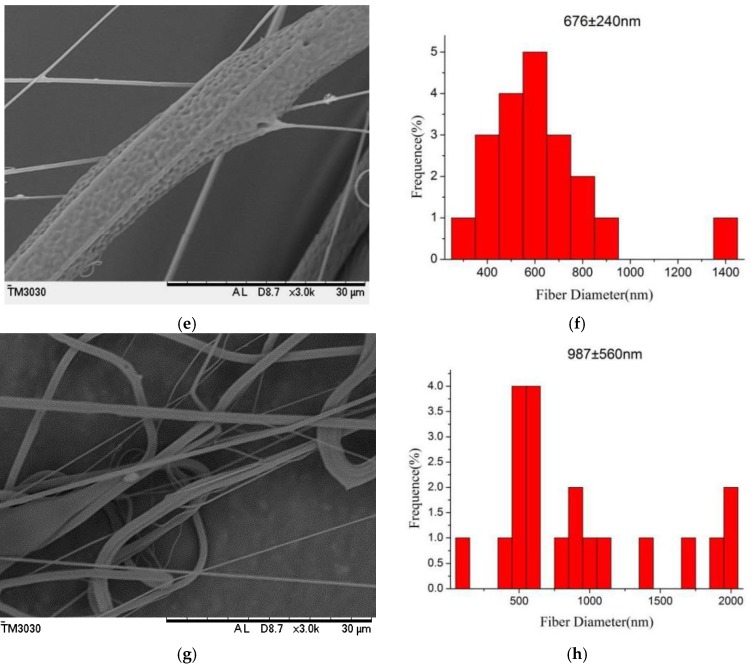
SEM micrographs and the diameter distribution histograms of nanoporous fibers via the airflow bubble-spun PLGA solutions with relative humidity of (**a**) 70%; (**c**) 75%; (**e**) 80%; (**g**) 85% (temperature: 25 °C, collecting distance: 18 cm, solutions concentration: 8 wt %); (**b**,**d**,**f**,**h**) show the diameter distribution histogram of PLGA nanoporous fibers shown in (**a**,**c**,**e**,**g**), respectively. The diameter distribution histogram data are representative of independent experiments and all data are given as means ± SD (*n* = 20).

**Figure 6:**
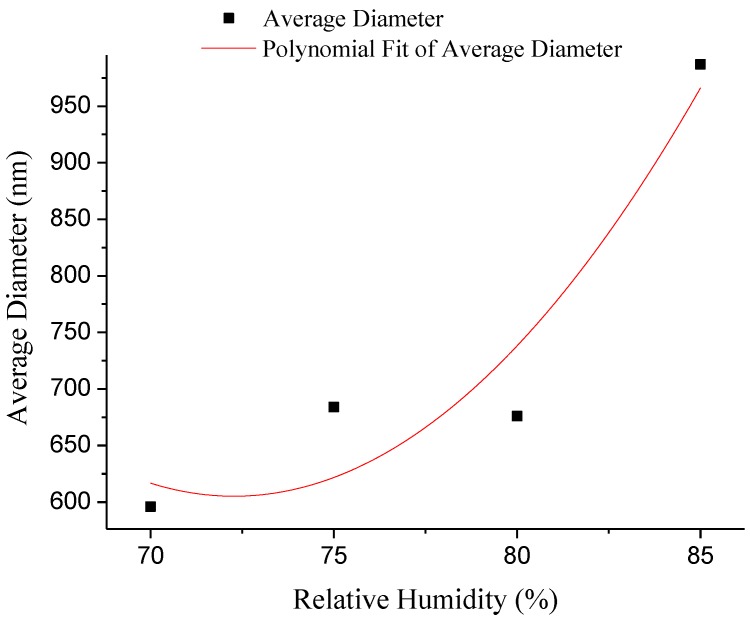
Average diameterof composite nanofibers with the different relative humidity.

The formation of porous fibers is mainly based on the high volatility of the solvent, and the relative humidity is also an important parameter for the control of the morphology of the fibers [31]. Figure 5 exhibits SEM micrographs and the diameter distribution histograms of nanoporous fibers via airflow bubble-spun PLGA solutions with different relative humidities (from 70% to 85%). As shown in Figure 5, when the relative humidity is greater than 70%, the fiber porosity is more obvious. However, with the volatilization of the solvent and the air flow caused by the blower, the relative humidity of the surroundings would change in an interval constantly. Through adjusting experiments, it was finally concluded that the spinning effect could be best when the relative humidity was controlled during the 70–80%. From Figure 5, it can be seen that the nanofibers can be produced at various relative humidities. The fiber diameters were found to be 596, 684, 676 and 987 nm at a relative humidity of 70%, 75%, 80% and 85% respectively. From Figure 3f, it can be seen that the average diameter of PLGA nanofibers was 527 nm at RH = 65%. From Figure 6, it can be seen that the diameter of the fibers increases non-linearly with the increase inrelative humidity.

### 3.3. Effect of MWCNTs on the Structure and Properties of PLGA Nanofiber Bundles Prepared by Airflow Bubble-Spinning

#### 3.3.1. Rheological Properties

**Figure 7:**
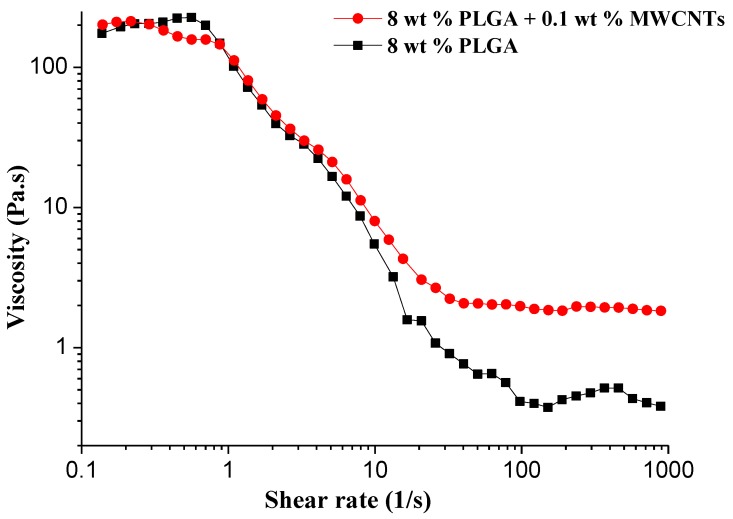
Rheological behavior between the shear viscosity and shear rate of the PLGA/MWCNT and PLGA solutions.

The viscosity plays a key role in airflow bubble-spinning since the viscous polymer bubble is stretched under the force of the airflow and ruptures to rush forward to the collecting plate. Therefore, it is necessary to study the effect of the addition of MWCNTs on the viscosity of the PLGA spinning solution. Figure 5 displays the rheological behavior between the shear viscosity and shear rate of the PLGA/MWCNT and PLGA solutions. To the best of our knowledge, the viscosity of the solutions was dependent on the concentration. The viscosity rose with an increase in the solution concentration. It has been reported that the orientation of macromolecular chains was the major cause of shear thinning behavior [29]. With the increase in the shear rate, the number of oriented polymer segments increased, which can decrease the viscosity, greatly promoting the shear thinning behavior. From Figure 7 we can see that the addition of MWCNTs has little effect on the viscosity of the 8 wt % PLGA solution.

#### 3.3.2. Morphology of the PLGA/MWCNT Composite Nanofibers

**Figure 8:**
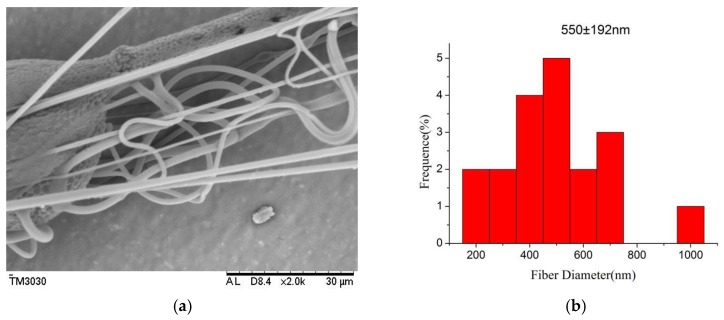
SEM micrographs and the diameter distribution histogramsof PLGA/MWCNTnanofibers (temperature: 25 °C, collecting distance: 18 cm, concentration: 8 wt %, relative humidity: 65%). (**b**) shows the diameter distribution histograms of PLGA/MWCNT nanoporous fibers shown in (**a**). The upper right corner in (**a**) isthe high-magnification image of the porous morphology. The diameter distribution histogram data are representative of independent experiments and all data are given as mean ± SD (*n* = 20).

The morphology of the PLGA/MWCNT composite nanofibers is shown in Figure 8. From Figure 8, it can be seen that the average diameter of the PLGA/MWCNT nanoporous fibers was found to be 550 nm. From Figure 3f, it can be seen that the average diameter of the PLGA nanofibers was 527 nm. The data illustrated that, after the addition of MWCNTs, the diameter of the nanofibers not only became slightly thicker but also more even. These results are consistent with the data from the literature [29].

#### 3.3.3. Thermo Gravimetric Analyzer (TGA)

**Figure 9:**
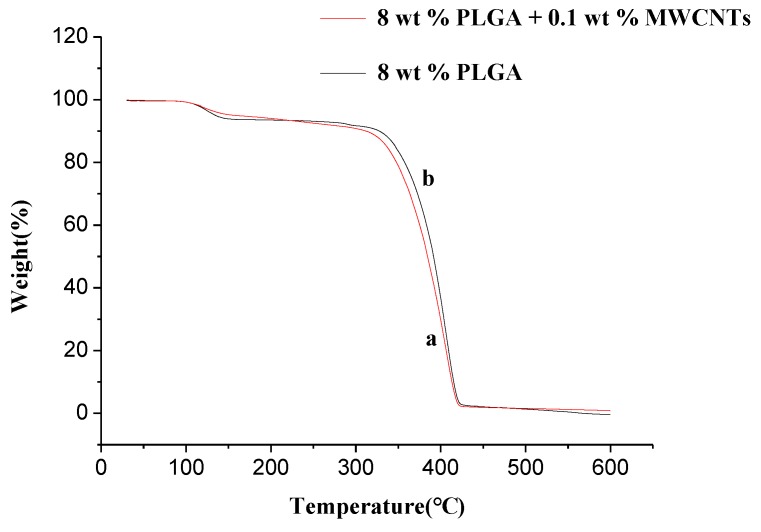
TGA profiles of PLGA/MWCNTs (**a**) and PLGA nanofibers (**b**) (temperature: 25 °C, collecting distance: 18 cm, concentration: 8 wt %, relative humidity: 65%).

The PLGA/MWCNTand PLGA nanofiberswere characterized using TGA (curves a and b in Figure 9). From Figure 9, it can be seen that the PLGA nanofibers begin to decompose at 325 °C and reach the maximum decomposition rate at 415 °C. When the temperature reach 420 °C, the residual mass becomes constant even if the temperature rises again. This means that the thermal decomposition of PLGA nanofibers was complete (Figure 9b). Compared to PLGA nanofibers, the PLGA/MWCNT composite nanofibers decompose at 330 °C and reach a maximum decomposition rate at 420 °C. Finally, the weight is kept constant,the same as for PLGA, at 420 °C. This shows that the multi-walled carbon nanotubes influence the thermal stability of PLGA, but the effect is weaker, which may be due to the small amount of MWCNTs that were added.

#### 3.3.4. X-ray Diffraction (XRD)

**Figure 10:**
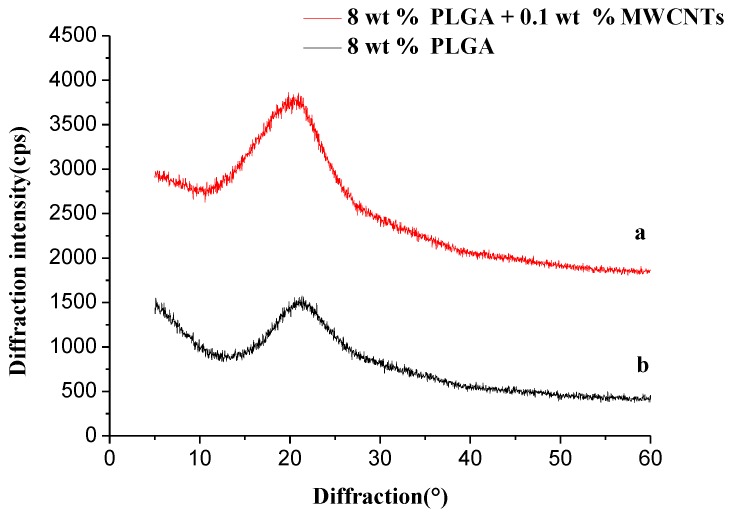
X-ray diffraction spectra of PLGA/MWCNTs (**a**) and PLGA nanofibers (**b**), respectively. (temperature: 25 °C, collecting distance: 18 cm, concentration: 8 wt %, humidity: 65%).

Figure 10 demonstrates the X-ray diffraction spectra of PLGA/MWCNTs and PLGA nanofibers. Many studies in the literature [32,33] show that the diffraction peaks of carbon nanotubes mainly occur around 26°. As shown in Figure 10, the diffraction peaks of pure PLGA mainly occur at 21°, while the diffraction peaks of PLGA/MWCNTs are mainly at 21.5°. The positions of the two diffraction peaks are basically the same, which further shows that there is almost no bonding reaction between PLGA and MWCNTs in PLGA/MWCNT nanofibers, and both of the components are physically combined.

#### 3.3.5. Fourier-Transform Infrared (FTIR) Spectroscopy

**Figure 11:**
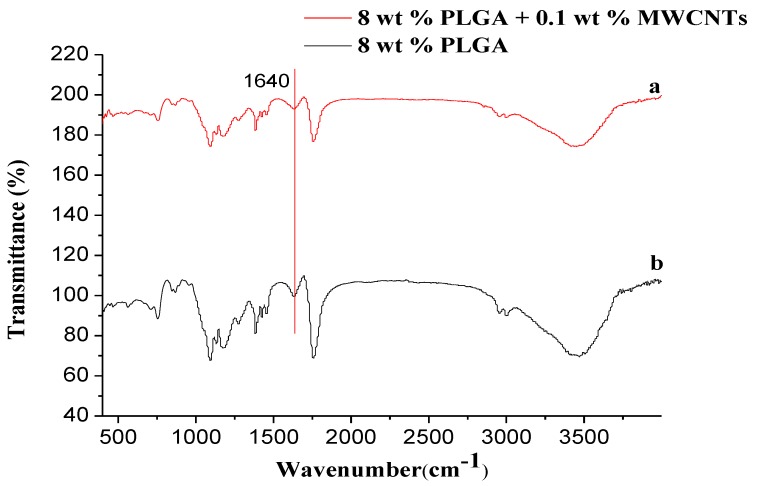
Infrared spectra of PLGA/MWCNT nanofibers (**a**) and PLGA nanofibers (**b**), respectively. (temperature: 25 °C, collecting distance: 18 cm, concentration: 8 wt %, humidity: 65%).

Figure 11 exhibits the infrared spectra of PLGA/MWCNT nanofibers (a) and PLGA nanofibers (b). As shown in Figure 11, below, the absorption peak corresponding to the carbon nanotube skeleton is the C=C contraction peak at 1640 cm^−1^, but due to the PLGA also having a C=C bond, s there is a superposition between these two peaks, which results in the presence of MWCNTs in the infrared spectrum is invisible observation.

In Figure 11, it can be see that there is no difference in peak position and peak intensity, and no new chemical bonds are formed, because both the MWCNT and PLGA macromolecules in the fibers are the only physical combination. This result is in agreement with the XRD data.

#### 3.3.6. Mechanical Behavior

**Figure 12:**
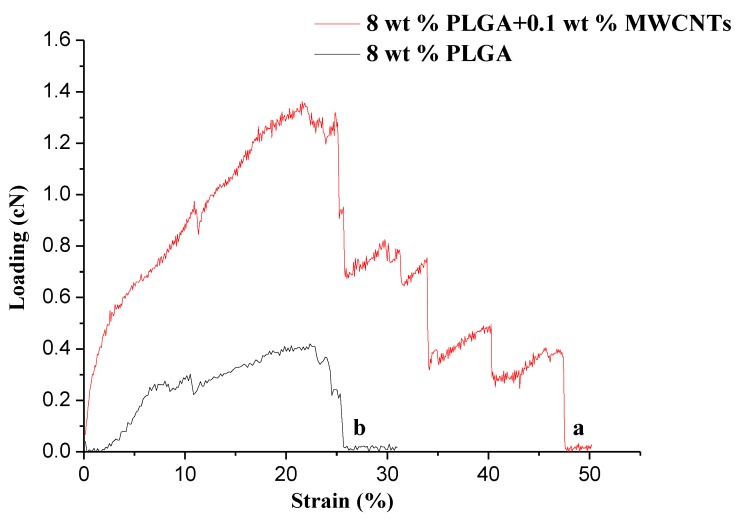
Load-strain curves for PLGA/MWCNTs (**a**) and PLGA (**b**)nanofibers, respectively.

In a series of experimental verifications, it was found that the addition of carbon nanotubes can significantly improve the mechanical properties of the composites [29,34]. We expected that PLGA/carbon nanotube composite nanofibers will display excellent mechanical properties. Figure 12 shows the load-strain curves of PLGA/MWCNT composite nanofibers (a) and PLGA nanofibers (b). As shown in Figure 12, they are the typical load-strain curves of PLGA nanofibers with and without MWCNTs. From Table 1, it can be clearly seen that the breaking strength and the elongation at the break of the composite nanofibers were significantly enhanced even with only 0.1 wt% MWCNTs mixed with the PLGA. In addition, the composite PLGA/MWCNT nanofibers exhibited a much higher Young’s modulus (the slope of the initial straight-line part in Figure 12a,b) when compared to that of pure PLGA nanofibers. The enhanced mechanical properties of the composite PLGA/MWCNT nanofiber should be ascribed to the presence of MWCNTs uniformly dispersed in the PLGA matrix. MWCNTs also have a large length to diameter ratio, and are flexible. When they are well dispersed in the polymer and the interface bonding strength is appropriate and there is no agglomeration, they will be able to achieve the effect of enhancing the mechanical properties of the fibers.

## 4. Conclusions

In conclusion, we systematically studied the effect of solution concentration, relative humidity, and spinning composition on the morphology of PLGA nanofibers fabricated by airflow bubble-spinning. PLGA or PLGA/MWCNT composite nanofibers with a porous structure were successfully obtained. We found that under certain constant spinning conditions, the concentration of the solution does not obviously affect the morphology of the fiber. However, the concentration will affect the diameter distribution of the obtained fibers. Based on the continuous optimization of the experimental parameters, nanoporous fibers can be formed. A better interconnected porous structure forms in a certain range and with greater relative humidity. In addition, under the optimized conditions, the formed PLGA/MWCNT composite nanofibers with relative uniform diameter distribution exhibited noticeably superior mechanical properties to those of pure PLGA nanofibers. The PLGA and PLGA/MWCNT composite nanofibers could be used for the future development of the fields of adsorption and filtration, which is currently ongoing in our laboratory.

## Figures and Tables

**Table 1:** Mechanical properties of PLGA/MWCNTs and PLGA nanofibers.

Sample	Breaking Strength (cN)	Elongation at Break (%)
PLGA/MWCNTs	1.36 ± 0.32	43.18 ± 6.22
PLGA	0.42 ± 0.17	22.35 ± 4.56
